# COVID-19 vaccine acceptance among adults in four major US metropolitan areas and nationwide

**DOI:** 10.1038/s41598-021-00794-6

**Published:** 2021-11-04

**Authors:** Ayman El-Mohandes, Trenton M. White, Katarzyna Wyka, Lauren Rauh, Kenneth Rabin, Spencer H. Kimball, Scott C. Ratzan, Jeffrey V. Lazarus

**Affiliations:** 1 Graduate School of Public Health & Health Policy, City University of New York (CUNY), New York, NY USA; 2 Barcelona Institute for Global Health (ISGlobal), Hospital Clínic, University of Barcelona, Barcelona, Spain; 3 Emerson College Polling, Emerson College, Boston, MA USA

**Keywords:** Health policy, Health services, Public health

## Abstract

This study assesses attitudes towards COVID-19 vaccination and the predictive value of COVID-VAC, a novel scale, among adults in the four largest US metropolitan areas and nationally. A 36-item survey of 6037 Americans was conducted in mid-April 2021. The study reports factors for COVID-19 vaccine acceptance among: (1) already vaccinated; (2) unvaccinated but willing to accept a vaccine; and (3) unvaccinated and unwilling to vaccinate. More than 20% were unwilling to vaccinate, expressing concerns about vaccine efficacy and safety and questioning the disease’s severity. Poverty, working outside of the home and conservative political views are predictors of unwillingness. Conversely, those who either personally tested positive for COVID-19, or had a family member who did so, were more likely to accept vaccination. Majorities of all respondents supported vaccination mandates for employees and university students. Respondents preferred to receive vaccines in their doctor´s office. Lower income and conservative ideology, but not race, were strongly associated with vaccine unwillingness. The predictive value of COVID-VAC was demonstrated. While vaccination mandates are likely to be accepted, additional effective, targeted interventions to increase vaccine uptake are needed urgently.

## Introduction

The COVID-19 pandemic continues to threaten population health, disrupt healthcare delivery and diminish economic and social activities globally, with over 230 million cumulative cases, 4.7 million deaths, and 6.2 billion doses of administered vaccines^1,2^. In the United States (US), there have been 44,314,424 reported COVID-19 cases and 716,847 deaths as of 1 October 2021, the highest number of deaths of any country in the world^3^. COVID-19 vaccinations in the US started on 13 December 2020, and 392,909,995 doses have been administered to date; 184,601,450 (55.5% of the population) are fully vaccinated^4,5^. Although 77% of American adults have now received at least one dose of a COVID-19 vaccine, trends indicate slowing rates and missing second shots^4^. New studies of evolving attitudes and opinions of Americans in different parts of the US who remain unwilling or uncertain about vaccination can contribute to strategic guidance for policymakers and healthcare providers.

Encouraging uptake of a COVID-19 vaccine among the remaining unvaccinated will largely depend on motivating those expressing a degree of vaccine hesitancy, defined by the WHO SAGE Working Group on Vaccine Hesitancy as the “delay in acceptance or refusal of vaccination despite availability of vaccination services^6^.” Heterogenous beliefs and behaviours with respect to risk, health, and trust in authorities within and across countries influence individuals’ decisionmaking about vaccines^7^. Major factors associated with vaccine hesitancy include perceptions on risk, safety, efficacy and trust, in addition to sociodemographic characteristics^8–14^. In the United States, COVID-19 vaccine hesitancy has been influenced by these identified factors^15,16^ as well as political factors^14,17–19^.

Only two formal studies of changing US attitudes towards COVID-19 vaccine acceptance were published over the first five months of vaccine availability^20,21^. Earlier research on willingness to vaccinate, conducted while the vaccines were in development, showed public confidence rising and falling based on factors such as media coverage, political identity and trust in public health authorities^16,19,22–27^, as well as trust in government^28^. A December 2020 rapid systematic review concluded that achieving herd immunity hinged on research-based strategies to communicate vaccine risks and benefits and to target politically, demographically, and socio-economically diverse groups across the US^29^. Since then, numerous polls have measured vaccine attitudes and factors impacting uptake. The Kaiser Family Foundation’s monthly vaccine monitor confirmed that overall COVID-19 vaccine confidence continued to rise as vaccinations rolled out. Amongst those uncertain or against a COVID-19 vaccine, no key sentiment or sub-group (e.g. political, socioeconomic, demographic) was monolithic^30^.

This study explores factors associated with vaccine acceptance in the US in 2021, one year after the pandemic was declared, including perceptions of risk, safety, efficacy and trust in government as well as sociodemographic and political factors. It also tests the predictive value of a novel 6-item vaccine acceptance scale, COVID-VAC. Respondents’ motivators and barriers, preferred sources of information and vaccination location were explored to help inform vaccination strategies.

## Results

### Study population

The four US metropolitan areas had a higher percentage of racial and ethnic minority respondents than the national sample (Table 1, Supplemental Table S1). Educational levels (bachelor’s degree and above) and income levels (US $75,000 and above) were also higher in the metropolitan areas. In general, a higher percentage of respondents in the metropolitan areas worked outside the home than found in the national sample. While political views were represented in relatively equal proportions nationally, metropolitan areas with the exception of Dallas (22%) had significantly fewer respondents with conservative views (13–17.2%).

**Table 1:** Demographics and COVID-19 vaccine intention or uptake in each of the five samples.

	National		Metropolitan area											
			NY		LA		Dallas		Chicago		NY	LA	Dallas	Chicago
	n	%	n	%	n	%	n	%	n	%	*P*-value			
**Age**														
18–29	402	19.9	207	20.5	225	22.5	230	23	209	20.8	0.997	0.929	0.032	0.976
30–39	349	17.3	181	17.9	188	18.7	203	20.3	183	18.2				
40–49	333	16.5	168	16.7	163	16.2	190	18.9	170	16.9				
50–59	341	16.9	168	16.7	163	16.3	162	16.2	170	16.9				
60–69	301	14.9	142	14.1	138	13.8	122	12.2	144	14.3				
70 +	295	14.6	142	14.1	125	12.5	95	9.5	131	13				
**Sex**														
Male	974	48.2	464	46	476	47.6	479	47.8	477	47.4	0.158	0.739	0.834	0.270
Female	1012	50.1	504	50.1	496	49.6	499	49.8	497	49.4				
Prefer not to say/other	34	1.7	39	3.9	28	2.8	24	2.4	32	3.2				
**Race**														
White	1221	60.4	448	44.5	291	29.1	453	45.2	519	51.6	< .0001	< .0001	< .0001	0.151
Black	248	12.3	159	15.8	60	6	161	16.1	160	15.9				
Latino/a	371	18.4	249	24.7	452	45.1	292	29.1	230	22.8				
Asian	109	5.4	110	10.9	161	16	70	7	70	6.9				
Other	70	3.5	150	14.9	41	4.1	26	2.6	28	2.8				
**Highest level of education**														
High school degree or less	772	38.2	379	37.6	390	39	364	36.4	349	34.7	< .0001	0.310	0.491	0.117
Some college	578	28.6	209	20.8	260	26	273	27.3	259	25.7				
Bachelor's Degree	410	20.3	239	23.8	230	23	233	23.2	239	23.8				
Graduate degree or more	259	12.8	179	17.8	120	12	132	13.1	160	15.8				
**Household income**														
< $25,000	303	15	150	14.9	148	14.8	112	11.2	103	10.2	< .0001	< .0001	< .0001	< .0001
$25,000-$74,999	889	44	322	31.9	374	37.4	351	35	308	30.6				
$75,000-$150,000	500	24.7	241	23.9	238	23.8	295	29.4	258	25.7				
> $150,000	136	6.7	131	13	143	14.3	123	12.3	133	13.2				
Prefer not to say	192	9.5	164	16.3	98	9.8	121	12.1	205	20.3				
**Employment**														
Working from home	743	36.8	332	32.9	295	29.5	327	32.6	338	33.6	0.367	0.054	< .0001	0.225
Working outside the home	557	27.6	286	28.4	365	36.4	412	41.1	342	33.9				
Unemployed	719	35.6	389	38.6	342	34.1	263	26.3	327	32.5				
**Political views**														
Liberal	666	33	304	30.2	362	36.1	307	30.7	287	28.5	< .0001	< .0001	0.041	< .0001
Moderate	592	29.3	355	35.2	346	34.5	344	34.4	408	40.5				
Conservative	579	28.6	176	17.4	131	13	225	22.5	158	15.7				
Don’t know/ prefer not to answer	183	9.1	173	17.2	163	16.3	125	12.5	154	15.2				
**COVID -19**														
Positive test-self	220	10.9	179	17.8	102	10.1	158	15.8	89	8.8	0.068	0.532	0.071	0.416
Positive test-household member	219	10.9	90	9	145	14.5	135	13.5	85	8.4				
Both	60	3	31	3.1	46	4.6	46	4.6	43	4.3				
Negative test or not tested	1520	75.3	707	70.2	708	70.7	662	66.1	790	78.4				
**Geographic location**														
Rural	334	16.5	5	0.5	0	0	66	6.6	11	1.1	n/a	n/a	n/a	n/a
Non-Rural	1686	83.5	1002	99.5	1001	100	936	93.4	996	98.9				
Vaccine received (1 dose or 2 doses)	658	32.6	398	39.5	367	36.7	344	34.4	412	40.9	< .0001	< .0001	0.526	< .0001
Vaccine not received but planned	929	46	508	50.4	519	51.8	460	45.9	483	47.9				
Vaccine not received and not planned	433	21.4	101	10.1	115	11.5	197	19.7	112	11.2				

n, % are weighted to the geographic population; *P*-values are based on weighted chi-squared tests comparing each metropolitan areas to the national average.

### National and metropolitan area vaccination rates and intent to vaccinate

Vaccination rates were significantly (*P* < 0.001) higher in three metropolitan areas (Chicago, NY, LA) than either for Dallas or the national sample (Table 1). The top reasons to vaccinate were protection from COVID-19 for oneself (national 42.3%, metropolitan areas 38.5–50%; Table 2), or family and friends (national 30%, metropolitan areas 24.4–33.5%) and to “help end the pandemic” (national 23.4%, metropolitan areas 19.7–25.5%). More than a third of the respondents who had already received the vaccine said they encountered difficulties in making an appointment. The top reasons given were lack of proximity of available sites, or not knowing how to sign up for an appointment. Approximately half of the respondents not yet vaccinated still plan to do so. Of this group, a plurality indicated a preference for receiving the vaccine in their doctor’s office compared to other sites. The majority of respondents who have been vaccinated or plan to do so felt confident the vaccine would protect against all variants of COVID-19 (Supplemental Table S6).

**Table 2:** Vaccination reasons.

	National	Metropolitan area			
		NY	LA	Dallas	Chicago
	%	%	%	%	%
**Among vaccinated respondents**					
**Reasons to receive vaccine**					
I want to protect myself from COVID-19	42.3	45.5	38.5	36.2	50
I want to protect my friends and family from COVID-19	30	28.5	26.2	33.5	24.4
To help end the pandemic more quickly	23.4	21.3	25.2	25.5	19.7
I need it to get back to work	2	2.9	2.3	2.3	3.6
My doctor or health plan/insurance recommended it	2	1.4	7.8	2.2	2.2
**Difficulty in making an appointment to get the vaccine**					
Yes	36.2	43	30.3	36.6	42.8
**Reasons for difficulty**					
I didn’t know how to sign up for an appointment	23.5	32.4	36	31.8	23.8
I didn’t know if I was eligible for a vaccine	19.5	13.8	18.5	18.2	17.3
There were no vaccination sites close to me	30.5	44.9	30.8	30.2	36.4
I was worried about the cost	17.5	4.3	5.7	8.4	17.7
Other	5.8	4.5	8.9	11.4	4.9
**Among respondents not yet vaccinated**					
**Top reason for waiting**					
See how it works in other people	23.8	22.3	15.6	19.9	19.2
Let high-risk people go first	8.4	9.6	11.5	9.6	12.5
Wait until it is more convenient	9.3	2.9	8.9	3.6	1.9
Wait until I know there are no serious complications	58.5	65.2	64	66.9	66.4
**What would help get ready for vaccine**					
Nothing will change my mind	53.5	49.3	62.8	63.9	59.1
Assurance by a family member or close friend who got vaccinated	12.8	2	6	6.4	1.2
Advice from community/religious leaders	1.1	1.3	0.8	3.9	1.4
Recommendation from my family doctor	9	15.2	6.3	5.4	4.6
Endorsement from a trusted political leader	2.7		0.4	1.9	3.8
Something else	20.8	32.3	23.7	18.5	29.8
**What would be the reason to receive vaccine**					
I want to protect myself from COVID-19	16.3	12.1	9.7	17	7.3
I want to protect my friends and family from COVID-19	14.3	32.5	18.9	10.1	8.1
To help end the pandemic more quickly	11.1	6.7	9.6	9.2	14.9
I need it to get back to work	5.4	5.5	0.9	7.2	7
My doctor or health plan/insurance recommended it	5.1	5.3	3.9	2.2	10.6
I will not get the vaccine for any reason	47.8	38	57.1	54.2	52.2
**Where would you most prefer to get the vaccine?**					
Local pharmacy like CVS or Walgreens	15.6	23.1	11.4	16.4	16
Hospital	14.8	13.1	27.8	4.5	7.8
Sports stadium	1	0.7	0.6	0.2	0.6
Your doctor’s office	38.5	39.1	36.2	44.7	50.9
Mobile unit send by the department of health to your neighborhood	7.8	2.1	4.3	4.3	3
Local schools	1.6	3.7	1.4	0	0.2
At a shopping mall or community centers	0	1.6	0.4	0.5	0
My place of worship (e.g. church, temple, mosque)	1.7	0.7	0.4	1.2	4.1
Somewhere else	19	15.9	17.6	28.2	17.4

% are weighted to the geographic population.

More than one-fifth (21.4%) of the national respondents indicated an unwillingness to vaccinate (Table 1). With the exception of the Dallas metropolitan area (19.7%), unwillingness to vaccinate was significantly lower in NY (10.1%), LA (11.5%), and Chicago (11.2%) than the national average (*P* < 0.001). The main reason for unwillingness was “waiting to see if there are no serious complications” (Table 2). When asked what would increase their readiness to accept vaccination, almost half of those who expressed unwillingness said that “nothing would change their minds” (Table 2).

Unwillingness to vaccinate was significantly lower among respondents age 60 and older compared to younger respondents (*P* < 0.0001; Table 3) in the national sample, but was comparable among women and men, different racial groups and educational levels. Except in Dallas and Chicago, where findings were mixed, unwillingness to vaccinate was significantly greater among lower-income individuals (*P* < 0.001). Unwillingness was also uniformly greater among individuals with moderate (25.5%) to conservative (26.7%) political views, compared to political liberals (10.9%), a trend that held across all metropolitan areas and the national sample (*P* < 0.0001). Unwillingness also correlated significantly with whether the respondent worked from home, worked outside the home or was unemployed (*P* = 0.007), and whether the individual or a household member had not received a positive test for COVID-19 in the past, compared to having tested positive (*P* = 0.001). Compared to similar respondents from the other three metropolitan areas, both White and Black residents of the Dallas metropolitan area were significantly less willing to vaccinate, as were those with less than a college degree (*P* < 0.001).

**Table 3:** Unwillingness to vaccinate by sociodemographic factors.

	National	Metropolitan area								
		NY	LA	Dallas	Chicago		NY	LA	Dallas	Chicago
	%	%	%	%	%	aOR (95%CI) *P*-value	aOR (95%CI) *P*-value	aOR (95%CI) *P*-value	aOR (95%CI) *P*-value	aOR (95%CI) *P*-value
**Vaccine not received and not planned**	21.4	10.1	11.5	19.7	11.2	–	–	–	–	–
**Age**						< .0001	0.218	0.002	0.001	0.043
18–29	24.2	10.5	14	24.5	13	Ref	Ref	Ref	Ref	Ref
30–39	22.3	18.2	12.2	24.1	10	1.03 (0.59, 1.78)	1.41 (0.44, 4.54)	0.83 (0.16, 4.23)	1.20 (0.41, 3.49)	1.47 (0.26, 8.31)
40–49	29.4	11	10.6	22.7	12.6	1.18 (0.64, 2.16)	0.66 (0.15, 2.87)	0.49 (0.11, 2.07)	0.64 (0.22, 1.92)	2.42 (0.43, 13.55)
50–59	25.2	8.9	14.6	16.1	13.9	0.76 (0.40, 1.42)	1.03 (0.25, 4.23)	0.58 (0.17, 2.05)	0.49 (0.17, 1.40)	0.86 (0.15, 5.09)
60–69	17.5	6.6	11.4	11.4	8	0.38 (0.18, 0.80)	0.58 (0.16, 2.12)	0.16 (0.04, 0.71)	0.16 (0.05, 0.54)	0.37 (0.05, 2.68)
70 +	7.2	2.7	3.1	9.2	7.9	0.10 (0.04, 0.24)	0.24 (0.05, 1.17)	0.05 (0.01, 0.23)	0.14 (0.04, 0.56)	0.3 (0.05, 1.64)
**Sex**						0.271	0.031	0.569	0.118	0.572
Male	20.7	8.6	11.1	23.5	11.9	Ref	Ref	Ref	Ref	ref
Female	21.6	11.5	11.9	16.4	8.5	1.24 (0.85, 1.82)	2.41 (1.08, 5.36)	1.29 (0.54, 3.07)	0.60 (0.31, 1.14)	1.34 (0.49, 3.71)
**Race**						0.530	0.089	0.323	0.006	0.586
White	22.6	12.8	15.8	27.7	10	Ref	Ref	Ref	Ref	ref
Black	20.1	7.5	5.4	21.1	12	0.90 (0.52, 1.57)	0.61 (0.19, 2.03)	0.40 (0.08, 2.15)	1.10 (0.45, 2.73)	1.6 (0.44, 5.81)
Latino/a	19.3	9.5	8.2	8.3	9.9	0.64 (0.34, 1.22)	0.37 (0.09, 1.45)	0.51 (0.18, 1.46)	0.20 (0.07, 0.57	2.29 (0.62, 8.47)
Asian	17.6	1.6	11.7	4.7	9.6	0.72 (0.28, 1.88)	0.13 (0.02, 0.80)	0.41 (0.12, 1.38	0.08 (0.01, 0.81)	0.65 (0.1, 4.36)
**Highest level of education**						0.227	0.042	0.033	< .001	0.017
High school degree or less	24.8	8.7	7.8	24.3	9.5	Ref	Ref	Ref	Ref	Ref
Some college	23.7	14	18.3	22.4	12.9	0.93 (0.58, 1.50)	2.42 (0.75, 7.83)	1.83 (0.59, 5.62)	0.92 (0.36, 2.36)	1.67 (0.51, 5.50)
Bachelor's Degree	16.3	12.3	12.6	13.7	16	0.66 (0.42, 1.02)	1.53 (0.47, 5.04)	0.81 (0.22, 2.97)	0.28 (0.11, 0.74)	3.12 (0.93, 10.44)
Graduate degree or more	14.6	5.3	6.4	11.9	4.7	0.80 (0.47, 1.35)	0.49 (0.13, 1.85)	0.30 (0.06, 1.36)	0.18 (0.06, 0.57)	0.32 (0.05, 1.96)
**Household income**						< .0001	0.060	0.088	0.102	0.611
< $25,000	35.1	13.9	20.4	32.8	6.8	ref	ref	ref	Ref	Ref
$25,000-$74,999	21.5	8.4	6.6	13.3	9.8	0.41 (0.23, 0.72)	0.20 (0.05, 0.8)	0.13 (0.03, 0.57)	0.43 (0.12, 1.57)	0.91 (0.17, 4.85)
$75,000-$150,000	15.8	11	11.8	17.3	13.5	0.2 (0.16, 0.52)	0.37 (0.1, 1.33)	0.3 (0.07, 1.28)	0.55 (0.14, 2.08)	2.43 (0.38, 15.56)
> $150,000	14.2	6.6	12.5	21.2	12.7	0.20 (0.10, 0.42)	0.14 (0.02, 0.95)	0.44 (0.09, 2.17)	1.06 (0.26, 4.30)	1.61 (0.23, 11.48)
Prefer not to say	19.4	11.1	14.3	30.5	11.4	0.26 (0.10, 0.64)	0.08 (0.01, 0.58)	0.44 (0.09, 2.16)	1.96 (0.47, 8.20)	0.80 (0.09, 7.17)
**Employment**						0.007	0.081	0.632	< .001	0.854
Working from home	16.3	9.4	13.1	9	8.6	0.47 (0.27, 0.82)	3.44 (0.96, 12.39)	1.09 (0.32, 3.69)	0.59 (0.21, 1.62)	0.99 (0.31, 3.12)
Working outside the home	29	15.7	11.3	27.3	16.9	0.90 (0.53, 1.52)	3.47 (1.15, 10.50)	1.79 (0.43, 7.41)	3.02 (1.17, 7.80)	0.70 (0.17, 2.85)
Unemployed	20.8	6.4	10.3	21.1	7.7	Ref	Ref	Ref	Ref	Ref
**Political views**						< .0001	< .0001	< .001	< .0001	< .0001
Liberal	10.9	3.6	7.1	15.3	1.9	Ref	Ref	Ref		
Moderate	25.5	8.2	9.5	7.8	8.5	3.96 (2.34, 6.70)	3.01 (1.22, 7.47)	2.11 (0.73, 6.09)		
Conservative	26.7	19.3	24.6	37.9	22	5.42 (3.18, 9.21)	8.30 (2.98, 23.17)	9.12 (3.04, 27.37)		
Don’t know/ prefer not to answer	29.9	15.8	14.9	30.4	24.3	4.88 (2.22, 10.76)	-	-		
**COVID-19**						0.001	0.099	0.001	0.446	0.138
Positive test-self	9.4	9.8	0.6	8.4	12.9	0.33 (0.16, 0.67)	0.29 (0.08, 1.00)	0.03 (0.00, 0.27)		
Positive test- household member	17.0	7.1	4.4	21.7	4.7	0.54 (0.29, 1.02)	0.34 (0.05, 2.13	0.09 (0.01, 0.57)		
Both	12.3	1.9	6.0	19.3	23.8	0.25 (0.08, 0.74)	0.09 (0.01, 1.33)	0.61 (0.10, 3.93)		
Negative test or not tested	24.2	1.9	14.9	22.0	11.0	Ref	Ref	Ref		
**Geographic location**										
Rural	79.1	–				–	–	–	–	
Non-rural	78.5	–				–	–	–	–	

% are weighted to the geographic population; *P*-value is for Type 3 test of overall effects based on weighted logistic regression (outcome = unwillingness to vaccinate). In the Chicago metropolitan area model reference category for political view was changed to moderate due to small sample size in the liberal category.

*aOR* adjusted odds ratios, *CI* confidence intervals.

### COVID-19 vaccine acceptance scale (COVID-VAC)

The six-item vaccination acceptance scale, which contains items on perceptions of risk, trust, safety, and efficacy, was unidimensional and reliable. Its first component explained 62.8% of variance with the remaining factors having eigenvalues below 1 (Cronbach’s alpha = 0.87). The high vaccine acceptance score recorded on this scale indicates strong endorsement of the six statements about COVID-19 and vaccines. Individual scores correlated strongly with actual or planned vaccination nationally (OR = 10.16; 95% CI 7.01–14.71) and in the four metropolitan areas: NY (OR = 9.59; 95% CI 5.33–17.25), LA (OR = 6.43; 95% CI 3.13–13.23), Dallas (OR = 12.18; 95% CI 7.55–19.16), and Chicago (OR = 14.17; 95% CI 6.65–30.20). Scores were stable across demographic groups classified by age, gender, race, education and income level (Supplemental Table S2).

The statement that “COVID-19 is a dangerous health threat” (Table 4) generated the highest degree of agreement (nationally 84%, metropolitan areas 82.3–90.4%). However, more than a quarter (25.8%) of the national respondents did not believe that the dangers of COVID-19 exceed those of the vaccine, a level of skepticism found less frequently in the metropolitan areas. One in five (21.7%) national sample participants also disbelieved that COVID-19 can be prevented by vaccination (metropolitan areas 15.6–19.3%); more so among participants who reported holding conservative views (Supplemental Table S3). However, around three-fourths of responents did trust in the science behind the vaccines (72.9% nationally, metropolitan areas 72.7–85.2%, Table 4).

**Table 4:** COVID-VAC score and score components.

	National		Metropolitan areas							
			NY		LA		Dallas		Chicago	
	M	SD	M	SD	M	SD	M	SD	M	SD
COVID-19 vaccine acceptance scale (COVID-VAC)	4.08	0.91	4.30	0.76	4.21	0.83	4.06	0.97	4.23	0.85
Vaccine received	4.44	0.57	4.46	0.56	4.34	0.74	4.45	0.56	4.49	0.55
Vaccine not received but planned	4.36	0.60	4.45	0.54	4.42	0.53	4.36	0.56	4.40	0.57
Vaccine not received and not planned	2.91	0.92	2.95	0.98	2.87	0.98	2.66	1.04	2.57	0.89

	OR 95%CI		OR 95%CI		OR 95%CI		OR 95%CI		OR 95%CI	
	n	%	n	%	n	%	n	%	n	%
COVID -VAC as a predictor of unwillingness to vaccinate	10.16 (7.01, 14.71)		9.59 (5.33,17.25)		6.43 (3.13,13.23)		12.18 (7.75,19.16)		14.17 (6.65, 30.20)	
**COVID-19 is a dangerous health threat**										
Strongly agree/agree	1697	84	910	90.4	829	82.8	824	82.3	869	86.2
Unsure/disagree/strongly disagree	323	16	97	9.6	172	17.2	178	17.7	138	13.8
**COVID-19 can be prevented by vaccination**										
Strongly agree/agree	1581	78.3	850	84.4	824	82.3	80.7	80.7	83.6	83.6
Unsure/disagree/strongly disagree	439	21.7	157	15.6	177	17.7	19.3	19.3	16.4	16.4
**The risks of COVID-19 disease are greater than the risks of the vaccine**										
Strongly agree/Agree	1499	74.2	822	81.7	799	79.8	754	75.3	798	79.3
Unsure/Disagree/strongly disagree	521	25.8	185	18.3	202	20.2	248	24.7	209	20.7
**The COVID-19 vaccines available to me are safe**										
Strongly agree/agree	1506	74.5	830	82.4	843	84.2	745	74.3	785	77.9
Unsure/disagree/strongly disagree	514	25.5	177	17.6	158	15.8	257	25.7	222	22.1
**I trust that my government is able to deliver the COVID-19 vaccine to everyone, everywhere in my country, equally**										
Strongly agree/agree	1406	69.6	755	74.9	762	76.2	722	72.1	707	70.2
Unsure/disagree/strongly disagree	614	30.4	252	25.1	239	23.8	280	27.9	300	29.8
**I trust the science behind the COVID-19 vaccines**										
Strongly agree/agree	1473	72.9	858	85.2	788	78.8	728	72.7	820	81.4
Unsure/disagree/strongly disagree	547	27.1	149	14.8	213	21.2	274	27.3	187	18.6

% are weighted to the geographic population; COVID -VAC as a predictor of unwillingness to vaccinate was evaluated using weighted logistic regression (outcome = unwillingness to vaccinate). Original response options ranged from strongly agree = 1 to strongly disagree = 5 for the presented item.

*OR* odds ratios, *CI* confidence intervals.

A slightly lower but still solid majority believed that COVID-19 vaccines would be distributed fairly “to everyone and everywhere” (69.6% nationally, metropolitan areas 70.2–76.2%, Table 4). Greater doubts about fairness were found among those with lower incomes (35.7%), worked outside the home (38.3%), or did not state their political views (45.2%). However, these factors showed no consistent associations across the metropolitan areas (Supplemental Table S3).

### Vaccination requirements and documentation

The majority of respondents agreed that employers have the right to require vaccination (Supplemental Table S4). Findings were even stronger with respect to universities requiring students to be vaccinated, but less so with respect to government-required vaccination.

Respondents overall expressed the highest level of approval for requiring proof of vaccination for international travel. However, approval for this policy was substantially lower among respondents who were unwilling to vaccinate or who reported conservative political views (Supplemental Figure S1).

### Preferred vaccination locations among those not vaccinated

Amongst currently unvaccinated respondents, the doctor’s office was the preferred choice for receiving the vaccine. The local pharmacy was a distant second (Table 2).

### Sources of vaccine information

There was a high level of agreement that the CDC is the leading source of information about COVID-19 vaccination (Supplemental Table S5), followed at some distance by local health providers. The respondents’ preferred media for obtaining COVID-19 information were cable TV and local news. This finding was consistent across racial and ethnic groups (Data not shown in table).

### Top priorities and rationales for vaccination

Among respondents who were vaccinated or planned to do so, the top priority was “getting everyone vaccinated as soon as possible” (Supplemental Table S5), followed by “getting children back to school” and “getting people back to work”. Among the minority of respondents who said they were unwilling to vaccinate, the top priority for them to even consider doing so was “getting people back to work”.

## Discussion

This study on COVID-19 vaccine acceptance in the United States found sharp contrasts in vaccination willingness among unvaccinated respondents in the national sample compared to the four metropolitan areas studied. Unwillingness to vaccinate was not uniformly distributed by age or racial group in any of the five populations surveyed. Those expressing COVID-19 vaccine hesitancy were unlikely to change their mind, most likely to have less education and lower income, and to work outside of the home. Respondents who reported that they or another household member had tested positive for COVID-19 were strongly motivated to accept vaccination.

Recent studies report levels of COVID-19 vaccine hesitancy in the United States ranging from 22% to 42.4%^14,28,31,32^, similar to the range we report. One study reported even lower vaccination intent in the New York and Chicago regions for the Department of Health and Human Services^13^. Our finding that half of those respondents who expressed unwillingness to vaccinate said that “nothing would change their minds,” should be a serious concern. Reassurance from family members or recommendations from doctors were reported “unlikely” to change this opinion, a level of recalcitrance that must be addressed in future policies and interventions.

It is important to consider key factors that differentiate those who are vaccine hesitant compared to those who are adamantly opposed to vaccination. The literature on COVID-19 vaccine hesitancy identifies lower education, income, and age; low perceived threat of infection; racial differences (Black or Latino/a vs White or Asian); political affiliation; living in a rural or generally remote area; and low trust in government, health authorities and scientific sources as key associations^14,28,31,32^. COVID-19 vaccine-specific concerns, assessed in COVID-VAC below, suggest a higher perceived risk of complications from the vaccines^13,32^. This risk perception is further amplified by the relatively lower conviction of dangers of COVID-19 disease among the sub-cohort of vaccine resisters.

Poverty was most frequently associated with unwillingness to vaccinate, with the exception of the Chicago metropolitan area. Hesitancy was also high among those who work outside the home, some of whom continued to perform essential but low-wage work even after lockdown^33,34^. In the Dallas metropolitan area, unemployment was also associated with unwillingness to vaccination. Limited economic resources are associated with conflicting life priorities that prevent individuals from prioritizing vaccination. Workers outside the home who have not yet been infected may have developed a perception of low risk from COVID-19^14,17,35,36^.

Many sources cite lower average vaccine acceptance among Blacks compared to other racial and ethnic groups^22,37–39^. Recent polls, including this study, indicate a relative increase in COVID-19 vaccine acceptance among Black respondents, however^40^. Our results found no consistent racial patterns to vaccine hesitancy. In the NY and LA metropolitan areas, white respondents were more likely than Black respondents to express COVID-19 vaccine unwillingness. The reverse was true in the Chicago and Dallas metropolitan areas. These findings caution against demographic generalizations regarding vaccine acceptance, and emphasize the importance of assessing local context when tailoring interventions and messaging. Stereotyping racial attitudes could even lead to disenfranchisement of communities of color that are increasingly well-motivated to participate in vaccination programs^37,41,42^.

The primary motivators to get vaccinated were “protecting myself against COVID-19”, followed by “protecting my family and friends”; these elements should remain at the core of vaccine communications messages. Testing positive for COVID-19 was a particularly strong motivator of vaccine acceptance. Continuing to encourage testing will counter misconceptions that the spread of the pandemic is over. As people continue to test positive across age, race, and ideology, this may encourage them to accept vaccination as a necessary protection.

The four metropolitan areas were chosen to cover all four US geographical regions with a significant combined proportion of the US population, their combined numbers of COVID-19 cases as a percentage of the US total, and their disproportionate influence on the national economy, cultural and informational trends^43,44^. Their high population density, greater reliance on public transport and status as major hubs of vacation and business travel also put them at particular risk for pandemic spread. Residents of these metropolitan areas have higher educational attainment and median income than national averages, but share equal or higher levels of poverty and unemployment; their racial and ethnic mix tends to favor higher representation of people of color (Supplemental Table S1)^37,45,46^.

Greater acceptance of vaccination may be higher in the New York, Chicago and Los Angeles metropolitan areas due to the more liberal political leanings of their residents. The politicization of the COVID-19 vaccine and other response efforts is well documented in the United States^47,48^. Conservative political affiliation is strongly associated with COVID-19 vaccine hesitancy^18,49^ as well as for other prevention measures such as facemask use, physical distancing, and stay-at-home orders^50^. When evaluating a decision to vaccinate, US conservatives may undervalue the risk to physical health posed by COVID-19 in relation to other perceived risks^7,51^, such as violation of individual autonomy or misconceptions of risks posed by vaccination^52,53^. In our study, holding conservative political views was associated with higher hesitancy across all metropolitan areas, whereas in the national sample moderate and conservative respondents were almost equally unwilling to accept a vaccine. Conservative respondents were more likely to believe falsely that vaccination would not prevent COVID-19 (with the exception of the LA metropolitan area).

Effective motivation of those holding conservative views should link vaccine coverage with returning to work, the top priority expressed by respondents with conservative views. Support from vaccine advocates and media channels with conservative views may mitigate hesitancy among this audiences^54^.

COVID-VAC showed external and internal validity, and predicted (from six times as often in LA to ten times as often nationally) the likelihood of vaccine acceptance. Agreement that COVID-19 is a dangerous health threat was high nationally and in the four metropolitan areas, even among those unwilling to vaccinate; in contrast, trust in the government’s ability to distribute vaccines fairly was uniformly lower for all respondents. This scale can be used to predict vaccination behaviors and as a comparative tool.

General and COVID-19-specific mistrust in the government and concerns about vaccine safety and efficacy are commonly cited as drivers of vaccine hesitance^6,38,55,56^. Trust in government regulation of vaccine development has significant influence on public confidence in vaccine safety and efficacy^57^. However, although trust in public health authorities is often cited as a source of confidence, it may not always play a significant role in the decisions of some Black people to accept a COVID-19 vaccine^58^.

Respondents from all study settings were more likely to support required proof of vaccination for access to international travel than domestic indoor events. Respondents were also more likely to accept employer vaccination mandates than government ones. As expected, those holding conservative poltical views were least likely to accept employer mandates and even less so a government one^59^. Though each state has the authority, none currently requires adult immunization against any disease^60^, and only a few are considering COVID-19 vaccination mandates^61^.

Although states require routine childhood immunization^62^, a pattern of increased vaccine hesitancy has been observed over the past decade^63^. Colleges and universities typically require state-mandated childhood vaccines and often require additional vaccines, such as hepatitis A and meningitis B^64^. A relatively high number of respondents in this study agreed that university students should be required to take the COVID-19 vaccine, a finding that has not yet been explored in COVID-19 literature.

In a CDC survey conducted in May 2020, public support for stay-at-home orders and closure of non-essential businesses was higher in NY (86.7%) and Los Angeles, CA (81.5%) than the national average (79.5%), with similar proportions reporting that state restrictions were not too restrictive^65^. Our results indicate a similar high level of support for vaccination requirements in these metropolitan areas, suggesting that risk communication strategies emphasizing individual empowerment and civic responsibility may be effective^66,67^.

All respondents tended to choose the CDC as their preferred source of vaccine information. Vaccine hesitant respondents were also likely to select other options, such as community leaders and local and state governments, suggesting that deploying community and local political leaders could be an appropriate strategy to reach them^68,69^.

In our study, the preferred media for respondents to obtain vaccine information were the internet and social media, followed by cable and local news. Both preferences can be double-edged swords. The internet and social media are sources of misinformation and disinformation, most commonly promulgated by political conservatives, including anti-vaccine activists^70,71^. Cable and online news organizations often supplant sensational crises or political content^72^. For example, viewers of one cable news provider were persuaded to not comply with social distancing recommendations^73^. Those who are hesitant may be more receptive, not only to online claims of misinformation, but to sensationalised reports on adverse effects and vaccine risks. Trusted sources should take proactive roles in delivering balanced, accurate information in internet, social, cable news and local TV media.

Most respondents preferred to receive COVID-19 vaccination in their doctors’ offices, fewer at local pharmacies or hospitals. Very few chose sports arenas, places of worship or mobile units. Opportunities for vaccination may be missed with routine health visits when the doctor’s office is ill-equipped to vaccinate^74^. As the first wave of enthusiasts is vaccinated, there will be a substantial decline in demand for vaccinations outside the routine healthcare setting^75^. Moving forward, ensuring proper storage and administration capability at primary healthcare facilities may be the way to accommodate public preferences^76,77^.

This study provides an in-depth understanding of perceptions underlying COVID-19 vaccine acceptance in the US, and describes motivational factors that could be used to sway those who are unsure or opposed to vaccination. Lower income and conservative ideology were strongly associated with vaccine hesitancy, as well as mistrust in vaccine efficacy and safety and underestimation of the risk of COVID-19. Non-immunization among these populations may delay or prevent reaching the level of herd immunity required to end this pandemic. Effective, targeted interventions are needed urgently. The COVID-VAC tool could be applied to specific populations, for example vaccine hesitant and vulnerable groups, to inform and further refine policies and strategies domestically and globally.

## Methods

### Sampling frame

A sampling frame of 242,127,140 US residents 18 years and older was employed. More than 80% of the US population live in urban areas^78^ and in 2019 the four largest metropolitan statistical areas had an estimated total population of approximately 50.5 million, representing approximately 15% of the total US population^79^. Five stratified proportionate randomized population based samples were drawn, one for each of the four most populous metropolitan statistical areas in the US: Northeast (New York-Newark-Jersey City, New York-New Jersey-Pennsylvania), Midwest (Chicago-Naperville-Elgin, Illinois-Indiana-Wisconsin), West (Los Angeles-Long Beach-Anaheim, California) and South (Dallas-Fort Worth-Arlington, Texas), and a fifth nationwide sample covering urban, semi-urban and rural geographies.

### Survey instrument design

A 36-item instrument (Supplemental File S1) was created by an expert panel following a comprehensive literature review of COVID-19 vaccine acceptance studies^28^. The instrument included a 6-item COVID-VAC scale representing perceptions of risk, trust, safety, and efficacy. The domains were adapted from vaccine acceptance predictors identified in earlier general vaccine confidence studies^6,80–89^.

### Multi-mode data collection design

Personalized short message service (SMS) text messages sent to each respondent. included a link to the survey and an opt-out option. Respondents could reply to the text with any queries and live operators were available to follow up with participants, also via SMS.

Interactive voice response (IVR) aka robo-poll messages were sent to landlines using a voice recorded IVR platform. An online opt-in panel of respondents was provided by Consensus Strategies and utilized to offset deficiency in the sample design with younger participants.The platforms were available in English and Spanish. Data were collected April 10–14, 2021.

### Data analysis

Demographic weights, developed based on the 2019 American Community Survey single-year estimates^90^, were applied: self-identified gender (male, female, prefer not to say and “other” categories), age (18–29, 30–39, 40–49, 50–59, 60–69, 70 +); race and ethnicity (African American/Black, Asian, Caucasian/White, American Indian, Native Hawaiian, Hispanic, and multiple/other); education (High School or less, some college, college degree, post graduate); census regions were used for the four metropolitan statistical areas and the Northeast, South, Midwest and West census regions were used for the national study.

The study reports COVID-19 vaccine acceptance among the following self-reporting sub-groups: (1) already vaccinated; (2) unvaccinated but willing to accept a vaccine; and (3) unvaccinated and unwilling to vaccinate. Trends were analysed using weighted descriptive statistics and chi-squared tests in the four metropolitan areas compared to the national averages. Associations between unwillingness to vaccinate and sociodemographic and political factors were assessed using weighted logistic regressions.

The paper also examined the predictive value of COVID-VAC, a novel scale that averages the reversed scores for survey items 1–6, based on responses ranging from strongly agree = 1 to strongly disagree = 5. Dimensionality of the scale was assessed using a principal component analysis (PCA) and reliability using Cronbach’s alpha. Weighted logistic regressions were used to evaluate its predictive capacity of perceptions most associated with vaccine acceptance. Statistical analyses were conducted using SAS version 9.4. Statistical significance was set at ∝  = 0.05.

### Ethics statement

This study was approved by the Emerson College Institutional Review Board (protocol number 20-023-F-E6/12), original expiration date of 11 June 2021. A renewal request was accepted on 12 April 2021 to extend the expiration date to 11 June 2022. The online questionnaire was administered after obtaining respondents’ informed consent on the landing page, which contained information about the survey and the purpose of this project. To comply with ethical compensation standards, equitable compensation per survey was applied (approximately US$ 2 per completion). Respondents’ identities were verified using IP addresses or mobile phones to ensure that each participant was real and unique upon initial registration, though this data, nor any other personally-identifiable data, were stored, as recommended by the British Psychological Society Ethics Guidelines for Internet-mediated Research and in adherence to the principles of the Declaration of Helsinki.

## Supplementary Information

Supplementary Information.
