# Supplementary material for: COVID-19 vaccine acceptance among adults in four major US metropolitan areas and nationwide
Source: Sci Rep. 2021 Nov 4;11:21844. doi: 10.1038/s41598-021-00794-6 (PMC8569192; doi:10.1038/s41598-021-00794-6)
Supplement: Supplementary file 1 — Supplementary Information. [file 41598_2021_794_MOESM1_ESM.docx]

**Supplemental Material**

Supplemental Table S1: U.S. population characteristics, COVID-19 cases and deaths per 100k and vaccination rates in the sampled areas

Supplemental Table S2: COVID-19 vaccine acceptance scale (COVID-VAC) by sociodemographic factors

Supplemental Table S3: Selected COVID-VAC score components by sociodemographic factors (vaccination equity, risk and prevention against COVID-19)

Supplemental Table S4: Vaccination requirements and documentation

Supplemental Table S5: Sources of information on the vaccine and top priorities for the country by vaccination status

Supplemental Table S6: Vaccination protection against all variants of COVID-19

Supplemental Figure S1: Approval for vaccination requirements and documentation by a) vaccination status and b) political views

Supplemental File S1: Survey instrument in a) English and b) Spanish

**Supplemental Table S1: U.S. population characteristics, COVID-19 cases and deaths per 100k and vaccination rates in the sampled areas**

|  | U. S. | Metropolitan area | | | |
| --- | --- | --- | --- | --- | --- |
|  |  | NY | LA | Dallas | Chicago |
| **Population size** | 328,239,523 | 19,216,182 | 13,214,799 | 7,573,136 | 9,457,867 |
| **Population density (people per square mile)** | 92.8 | 2,874.5 | 2723.9 | 873.1 | 1,314.50 |
| **Race** (%)  White  Black or African American  American Indian and Alaska Native  Asian  Native Hawaiian and Other Pacific Islander  Other  Two or more races  Hispanic | 60  12  1  6  0  0  3  18 | 45  16  0  11  0  1  2  25 | 29  6  0  16  0  0  3  45 | 45  16  0  7  0  0  2  29 | 52  16  0  7  0  0  2  23 |
| **Education** (%)  No degree  High School  Some college  Bachelor’s  Post-grad  High school grad or higher  Bachelor’s degree or higher | 11  27  29  20  13  88.6  33.1 | 13  25  21  24  18  87.3  41.8 | 19  20  26  23  12  81  35 | 13  23  27  23  13  83  36 | 11  24  26  24  16  89.4  39.3 |
| **Median household income** ($) | 65,712 | 83,160 | 77,774 | 72,265 | 75,379 |
| **Poverty** (% below poverty line) | 12.3 | 11.6 | 12.4 | 10.5 | 10.6 |
| **Unemployment** (%) | 6.6 | 9.8 | 9.9 | 6.8 | 8.3 |
| **Uninsured** (%) | 8.7 | 7.2 | 9.6 | 15.6 | 6.8 |
| **Medicaid** (%) | 17.6 | 29.5 | 25.2 | 12.5 | 25.6 |
| **Vaccinated** (%, average over counties in CBSA) | 23.1 | 23 | 19.6 | 33.6 | 19.7 |
| **Incidence of COVID-19/100k**  **Cumulative COVID-19 cases/100k**  **Death rate of COVID-19 /100k**  **Cumulative COVID-19 cases/100k** | 147.5  9572.69  1.5  171.95 | 301.96  11031.09  3.32  278.04 | 50.88  11216  2.53  179.8 | 45.57  11027.58  1.56  167.08 | 178.47  9829.75  1.75  153.31 |
| **Eligibility for the vaccine at the time of the survey** | N/A | Age 30+ (March 30); Age 16+ (April 6) | Age 50+ until April 15, then 16+ | Age 16+ (March 29) | Age 16-64 with underlying conditions (March 29); Age 16+  (April 12), except Chicago (April 19) |
| Sources: American Community Survey 2019; Unemployment data from U.S. Bureau of Labor Statistics Feb 2021; Uninsured data from CDC COVID data tracker; Medicaid data from DataUSA.io; Vaccination data from COVID data tracker, except Dallas MA from Texas Department of State Health Services; COVID-19 data from the CDC data tracker (7-day average). Vaccination and incidence data as of 4/12/21-4/14/1/21, US data as of 4/15/21. CBSA=Census Core-Based Statistical Areas. | | | | | |
|  |  |  |  |  |  |
|  |  |  |  |  |  |

**Supplemental Table S2: COVID-19 vaccine acceptance scale (COVID-VAC) by sociodemographic factors**

|  | National | |  | Metropolitan area | | | | | | | | | | |
| --- | --- | --- | --- | --- | --- | --- | --- | --- | --- | --- | --- | --- | --- | --- |
|  |  | |  | NY | |  | LA | |  | Dallas | |  | Chicago | |
| **Age** | M | SD |  | M | SD |  | M | SD |  | M | SD |  | M | SD |
| 18-29 | 3.97 | 0.78 |  | 4.18 | 0.68 |  | 3.93 | 0.82 |  | 3.97 | 0.94 |  | 4.12 | 0.73 |
| 30-39 | 4.04 | 0.86 |  | 4.16 | 0.79 |  | 4.25 | 0.68 |  | 4.05 | 0.98 |  | 4.21 | 0.84 |
| 40-49 | 3.98 | 0.95 |  | 4.22 | 0.90 |  | 4.26 | 0.72 |  | 3.97 | 0.95 |  | 4.15 | 0.97 |
| 50-59 | 3.96 | 1.10 |  | 4.36 | 0.75 |  | 4.15 | 1.10 |  | 4.07 | 1.13 |  | 4.16 | 0.91 |
| 60-69 | 4.13 | 0.94 |  | 4.42 | 0.68 |  | 4.39 | 0.83 |  | 4.19 | 0.94 |  | 4.41 | 0.83 |
| 70+ | 4.44 | 0.70 |  | 4.57 | 0.62 |  | 4.49 | 0.63 |  | 4.29 | 0.76 |  | 4.44 | 0.74 |
| **Sex** |  |  |  |  |  |  |  |  |  |  |  |  |  |  |
| Male | 4.10 | 0.90 |  | 4.29 | 0.79 |  | 4.30 | 0.78 |  | 3.99 | 0.99 |  | 4.22 | 0.89 |
| Female | 4.07 | 0.91 |  | 4.35 | 0.72 |  | 4.14 | 0.87 |  | 4.14 | 0.94 |  | 4.30 | 0.73 |
| **Race** |  |  |  |  |  |  |  |  |  |  |  |  |  |  |
| White | 4.07 | 0.93 |  | 4.26 | 0.80 |  | 4.23 | 0.96 |  | 3.84 | 1.13 |  | 4.34 | 0.77 |
| Black | 4.02 | 0.83 |  | 4.36 | 0.69 |  | 4.09 | 0.64 |  | 4.15 | 0.67 |  | 4.24 | 0.63 |
| Latino/a | 4.10 | 0.95 |  | 4.34 | 0.74 |  | 4.23 | 0.81 |  | 4.33 | 0.69 |  | 4.08 | 0.97 |
| Asian | 4.22 | 0.61 |  | 4.48 | 0.45 |  | 4.26 | 0.64 |  | 4.52 | 0.62 |  | 4.34 | 0.76 |
| **Highest level of education** |  |  |  |  |  |  |  |  |  |  |  |  |  |  |
| High school degree or less | 4.08 | 0.87 |  | 4.37 | 0.72 |  | 4.19 | 0.79 |  | 3.95 | 1.04 |  | 4.28 | 0.85 |
| Some college | 3.98 | 0.98 |  | 4.11 | 0.80 |  | 4.09 | 0.93 |  | 4.04 | 1.00 |  | 4.12 | 0.82 |
| Bachelor's Degree | 4.13 | 0.89 |  | 4.26 | 0.76 |  | 4.29 | 0.80 |  | 4.15 | 0.86 |  | 4.13 | 0.93 |
| Graduate degree or more | 4.21 | 0.88 |  | 4.44 | 0.72 |  | 4.42 | 0.72 |  | 4.23 | 0.85 |  | 4.45 | 0.68 |
| **Household income** |  |  |  |  |  |  |  |  |  |  |  |  |  |  |
| < $25,000 | 4.04 | 0.87 |  | 4.38 | 0.70 |  | 4.07 | 0.72 |  | 3.90 | 1.17 |  | 4.08 | 0.76 |
| $25,000-$74,999 | 4.06 | 0.88 |  | 4.26 | 0.63 |  | 4.32 | 0.65 |  | 4.15 | 0.77 |  | 4.28 | 0.75 |
| $75,000-$150,000 | 4.12 | 0.94 |  | 4.35 | 0.72 |  | 4.05 | 0.96 |  | 4.14 | 0.95 |  | 4.25 | 0.89 |
| >$150,000 | 4.21 | 0.95 |  | 4.37 | 0.77 |  | 4.38 | 0.86 |  | 4.06 | 1.05 |  | 4.20 | 1.05 |
| Prefer not to say | 3.99 | 1.00 |  | 4.19 | 1.01 |  | 4.16 | 1.07 |  | 3.75 | 1.17 |  | 4.23 | 0.81 |
| **Employment** |  |  |  |  |  |  |  |  |  |  |  |  |  |  |
| Working from home | 4.17 | 0.81 |  | 4.29 | 0.74 |  | 4.29 | 0.74 |  | 4.30 | 0.72 |  | 4.23 | 0.81 |
| Working outside the home | 3.81 | 1.02 |  | 4.08 | 0.88 |  | 4.13 | 0.85 |  | 3.84 | 1.09 |  | 4.10 | 0.98 |
| Not employed | 4.18 | 0.88 |  | 4.48 | 0.61 |  | 4.24 | 0.87 |  | 4.11 | 0.98 |  | 4.36 | 0.70 |
| **Political views** |  |  |  |  |  |  |  |  |  |  |  |  |  |  |
| Liberal | 4.43 | 0.60 |  | 4.54 | 0.53 |  | 4.45 | 0.59 |  | 4.38 | 0.74 |  | 4.61 | 0.50 |
| Moderate | 4.09 | 0.80 |  | 4.34 | 0.67 |  | 4.16 | 0.92 |  | 4.36 | 0.60 |  | 4.26 | 0.77 |
| Conservative | 3.77 | 1.09 |  | 4.02 | 0.86 |  | 3.68 | 1.00 |  | 3.37 | 1.20 |  | 3.89 | 0.99 |
| Don’t know/ prefer not to answer | 3.69 | 1.06 |  | 4.09 | 0.98 |  | 4.22 | 0.71 |  | 3.68 | 1.07 |  | 3.80 | 1.04 |

**Supplemental Table S3. Selected COVID-VAC score components by sociodemographic factors (vaccination equity, risk and prevention against COVID-19)**

|  | National | | |  | | Metropolitan area | | | | | | | | | | | | | | | | | | | | | | |
| --- | --- | --- | --- | --- | --- | --- | --- | --- | --- | --- | --- | --- | --- | --- | --- | --- | --- | --- | --- | --- | --- | --- | --- | --- | --- | --- | --- | --- |
|  |  |  | |  | | NY | | | | |  | | LA | | | |  | | Dallas | | | | |  | Chicago | | | |
|  | %  Unsure/diagree | %  Agree | |  | | %  Unsure/diagree | | %  Agree | | |  | | %  Unsure/diagree | | %  Agree | |  | | %  Unsure/diagree | | | %  Agree | |  | %  Unsure/diagree | | | %  Agree |
| **COVID-19 can be prevented by vaccination.** | | | | | | | | | | | | | | | | | | | | | | | | | | | | |
| Age |  |  | |  | |  | |  | | |  | |  | |  | |  | |  | | |  | |  |  | | |  |
| 18-29 | 17.3 | 82.7 | |  | | 10.4 | | 89.6 | | |  | | 29.1 | | 70.9 | |  | | 17.9 | | | 82.1 | |  | 2.6 | | | 97.4 |
| 30-39 | 24.1 | 75.9 | |  | | 23.7 | | 76.3 | | |  | | 15 | | 85 | |  | | 18.8 | | | 81.2 | |  | 26.5 | | | 73.5 |
| 40-49 | 28.6 | 71.4 | |  | | 23 | | 77 | | |  | | 14.1 | | 85.9 | |  | | 23.2 | | | 76.8 | |  | 23 | | | 77 |
| 50-59 | 27.2 | 72.8 | |  | | 13.7 | | 86.3 | | |  | | 17.3 | | 82.7 | |  | | 22.5 | | | 77.5 | |  | 22.5 | | | 77.5 |
| 60-69 | 20.4 | 79.6 | |  | | 13.1 | | 86.9 | | |  | | 12.9 | | 87.1 | |  | | 20.1 | | | 79.9 | |  | 11.6 | | | 88.4 |
| 70+ | 12.4 | 87.6 | |  | | 8.8 | | 91.2 | | |  | | 11.8 | | 88.2 | |  | | 9.9 | | | 90.1 | |  | 12.7 | | | 87.3 |
| Sex |  |  | |  | |  | |  | | |  | |  | |  | |  | |  | | |  | |  |  | | |  |
| Male | 18.3 | 81.7 | |  | | 15.8 | | 84.2 | | |  | | 12.4 | | 87.6 | |  | | 19.2 | | | 80.8 | |  | 16.5 | | | 83.5 |
| Female | 24.7 | 75.3 | |  | | 15.8 | | 84.2 | | |  | | 23.3 | | 76.7 | |  | | 18.8 | | | 81.2 | |  | 15.4 | | | 84.6 |
| Race |  |  | |  | |  | |  | | |  | |  | |  | |  | |  | | |  | |  |  | | |  |
| White | 21.3 | 78.7 | |  | | 11.9 | | 88.1 | | |  | | 19 | | 81 | |  | | 26.9 | | | 73.1 | |  | 10 | | | 90 |
| Black | 26.2 | 73.8 | |  | | 25.9 | | 74.1 | | |  | | 8.8 | | 91.2 | |  | | 12.8 | | | 87.2 | |  | 23.2 | | | 76.8 |
| Latino/a | 22.4 | 77.6 | |  | | 19.3 | | 80.7 | | |  | | 16.1 | | 83.9 | |  | | 11.3 | | | 88.7 | |  | 22.6 | | | 77.4 |
| Asian | 15 | 85 | |  | | 16.1 | | 83.9 | | |  | | 6.1 | | 93.9 | |  | | 20.7 | | | 79.3 | |  | 4.5 | | | 95.2 |
| Highest level of education |  |  | |  | |  | |  | | |  | |  | |  | |  | |  | | |  | |  |  | | |  |
| High school degree or less | 22.4 | 77.6 | |  | | 16.8 | | 83.2 | | |  | | 17 | | 83 | |  | | 20.6 | | | 79.4 | |  | 17.5 | | | 82.5 |
| Some college | 24.1 | 75.9 | |  | | 18.5 | | 81.5 | | |  | | 26.2 | | 73.8 | |  | | 19.1 | | | 80.9 | |  | 20.9 | | | 79.1 |
| Bachelor's Degree | 19.3 | 80.7 | |  | | 15.1 | | 84.9 | | |  | | 14.3 | | 85.7 | |  | | 20.6 | | | 79.4 | |  | 15.3 | | | 84.7 |
| Graduate degree or more | 18.4 | 81.6 | |  | | 10.3 | | 89.7 | | |  | | 8.2 | | 91.8 | |  | | 14.2 | | | 85.8 | |  | 8.2 | | | 91.8 |
| Household income |  |  | |  | |  | |  | | |  | |  | |  | |  | |  | | |  | |  |  | | |  |
| < $25,000 | 28.3 | 71.7 | |  | | 23.1 | | 76.9 | | |  | | 15.9 | | 84.1 | |  | | 32.4 | | | 67.6 | |  | 12.3 | | | 87.7 |
| $25,000-$74,999 | 20.3 | 79.7 | |  | | 14.1 | | 85.9 | | |  | | 15.1 | | 84.9 | |  | | 13.9 | | | 86.1 | |  | 16 | | | 84 |
| $75,000-$150,000 | 17.8 | 82.2 | |  | | 14.7 | | 85.3 | | |  | | 26.8 | | 73.2 | |  | | 13.2 | | | 86.8 | |  | 12 | | | 88 |
| >$150,000 | 19.1 | 80.9 | |  | | 14.3 | | 85.7 | | |  | | 9.7 | | 90.3 | |  | | 23.8 | | | 76.2 | |  | 19.6 | | | 80.4 |
| Prefer not to say | 30 | 70 | |  | | 13.9 | | 86.1 | | |  | | 20.2 | | 79.8 | |  | | 33.1 | | | 66.9 | |  | 22.3 | | | 77.7 |
| Employment |  |  | |  | |  | |  | | |  | |  | |  | |  | |  | | |  | |  |  | | |  |
| Working from home | 15.7 | 84.3 | |  | | 13.1 | | 86.9 | | |  | | 13.3 | | 86.7 | |  | | 10.9 | | | 89.1 | |  | 13 | | | 87 |
| Working outside the home | 31.9 | 68.1 | |  | | 21.5 | | 78.5 | | |  | | 18.1 | | 81.9 | |  | | 25.9 | | | 74.1 | |  | 17.5 | | | 82.5 |
| Not employed | 20.1 | 79.9 | |  | | 13.3 | | 86.7 | | |  | | 21.2 | | 78.8 | |  | | 19.6 | | | 80.4 | |  | 18.6 | | | 81.4 |
| Political views |  |  | |  | |  | |  | | |  | |  | |  | |  | |  | | |  | |  |  | | |  |
| Liberal | 10.1 | 89.9 | |  | | 7 | | 93 | | |  | | 10.6 | | 89.4 | |  | | 10.7 | | | 89.3 | |  | 5.5 | | | 94.5 |
| Moderate | 22.3 | 77.7 | |  | | 15.6 | | 84.4 | | |  | | 21.5 | | 78.5 | |  | | 6 | | | 94 | |  | 16.5 | | | 83.5 |
| Conservative | 29.1 | 70.9 | |  | | 22.9 | | 77.1 | | |  | | 32.3 | | 67.7 | |  | | 39.1 | | | 60.9 | |  | 20 | | | 80 |
| Don’t know/ prefer not to answer |  |  | |  | |  | |  | | |  | |  | |  | |  | |  | | |  | |  |  | | |  |
| **The risks of COVID-19 disease are greater than the risks of the vaccine.** | | | | | | | | | | | | | | | | | | | | | | | | |  |  |  |  |
| Age |  |  | |  | |  | |  | | |  | |  | |  | |  | |  | | |  | |  |  | | |  |
| 18-29 | 33.4 | 66.6 | |  | | 19.5 | | 80.5 | | |  | | 26 | | 74 | |  | | 24.4 | | | 75.6 | |  | 17.4 | | | 82.6 |
| 30-39 | 26.2 | 73.8 | |  | | 23 | | 77 | | |  | | 12.6 | | 87.4 | |  | | 23.1 | | | 76.9 | |  | 17.2 | | | 82.8 |
| 40-49 | 27 | 73 | |  | | 17.7 | | 82.3 | | |  | | 33.7 | | 66.3 | |  | | 36.4 | | | 63.6 | |  | 29 | | | 71 |
| 50-59 | 24.2 | 75.8 | |  | | 19 | | 81 | | |  | | 17.3 | | 82.7 | |  | | 17.8 | | | 82.2 | |  | 23.6 | | | 76.4 |
| 60-69 | 24 | 76 | |  | | 18.5 | | 81.5 | | |  | | 20.2 | | 79.8 | |  | | 21.7 | | | 78.3 | |  | 18.8 | | | 81.2 |
| 70+ | 17.4 | 82.6 | |  | | 10.6 | | 89.4 | | |  | | 7.3 | | 92.7 | |  | | 21.2 | | | 78.8 | |  | 18.4 | | | 81.6 |
| Sex |  |  | |  | |  | |  | | |  | |  | |  | |  | |  | | |  | |  |  | | |  |
| Male | 26.2 | 73.8 | |  | | 18 | | 82 | | |  | | 17.7 | | 82.3 | |  | | 28.2 | | | 71.8 | |  | 24.8 | | | 75.2 |
| Female | 25.1 | 74.9 | |  | | 17.8 | | 82.2 | | |  | | 22.8 | | 77.2 | |  | | 21.4 | | | 78.6 | |  | 14.9 | | | 85.1 |
| Race |  |  | |  | |  | |  | | |  | |  | |  | |  | |  | | |  | |  |  | | |  |
| White | 23.8 | 76.2 | |  | | 17.5 | | 82.5 | | |  | | 20.1 | | 79.9 | |  | | 32.4 | | | 67.6 | |  | 13.2 | | | 86.8 |
| Black | 30.3 | 69.7 | |  | | 26.2 | | 73.8 | | |  | | 26.8 | | 73.2 | |  | | 11.7 | | | 88.3 | |  | 23.7 | | | 76.3 |
| Latino/a | 33.2 | 66.8 | |  | | 15.6 | | 84.4 | | |  | | 20.7 | | 79.3 | |  | | 22.3 | | | 77.7 | |  | 32.7 | | | 67.3 |
| Other | 18.2 | 81.8 | |  | | 17 | | 83 | | |  | | 17 | | 83 | |  | | 17.6 | | | 82.4 | |  | 27.6 | | | 72.4 |
| Highest level of education |  |  | |  | |  | |  | | |  | |  | |  | |  | |  | | |  | |  |  | | |  |
| High school degree or less | 27.9 | 72.1 | |  | | 19.8 | | 80.2 | | |  | | 25.3 | | 74.7 | |  | | 26 | | | 74 | |  | 20.8 | | | 79.2 |
| Some college | 29.6 | 70.4 | |  | | 24.2 | | 75.8 | | |  | | 20.6 | | 79.4 | |  | | 29 | | | 71 | |  | 26.5 | | | 73.5 |
| Bachelor's Degree | 21.2 | 78.8 | |  | | 16.3 | | 83.7 | | |  | | 16.4 | | 83.6 | |  | | 23.5 | | | 76.5 | |  | 20.4 | | | 79.6 |
| Graduate degree or more | 18.6 | 81.4 | |  | | 11.2 | | 88.8 | | |  | | 10.1 | | 89.9 | |  | | 14.2 | | | 85.8 | |  | 11.7 | | | 88.3 |
| Household income |  |  | |  | |  | |  | | |  | |  | |  | |  | |  | | |  | |  |  | | |  |
| < $25,000 | 27.3 | 72.7 | |  | | 29.9 | | 70.1 | | |  | | 20.2 | | 79.8 | |  | | 29.9 | | | 70.1 | |  | 23.6 | | | 76.4 |
| $25,000-$74,999 | 25.5 | 74.5 | |  | | 12.9 | | 87.1 | | |  | | 18.3 | | 81.7 | |  | | 23.3 | | | 76.7 | |  | 19 | | | 81 |
| $75,000-$150,000 | 28.4 | 71.6 | |  | | 19.8 | | 80.2 | | |  | | 26.3 | | 73.7 | |  | | 20.5 | | | 79.5 | |  | 23.4 | | | 76.6 |
| >$150,000 | 14.2 | 85.8 | |  | | 12.2 | | 87.8 | | |  | | 16.8 | | 83.2 | |  | | 23.6 | | | 76.4 | |  | 18.7 | | | 81.3 |
| Prefer not to say | 26.2 | 73.8 | |  | | 21.3 | | 78.7 | | |  | | 17.4 | | 82.6 | |  | | 35.3 | | | 64.7 | |  | 19.8 | | | 80.2 |
| Employment |  |  | |  | |  | |  | | |  | |  | |  | |  | |  | | |  | |  |  | | |  |
| Working from home | 22.7 | 77.3 | |  | | 17.8 | | 82.2 | | |  | | 14.4 | | 85.6 | |  | | 18.6 | | | 81.4 | |  | 21 | | | 79 |
| Working outside the home | 34.7 | 65.3 | |  | | 23.5 | | 76.5 | | |  | | 24.4 | | 75.6 | |  | | 25.4 | | | 74.6 | |  | 26.5 | | | 73.5 |
| Not employed | 22.1 | 77.9 | |  | | 15 | | 85 | | |  | | 20.6 | | 79.4 | |  | | 31.2 | | | 68.8 | |  | 14.3 | | | 85.7 |
| Political views |  |  | |  | |  | |  | | |  | |  | |  | |  | |  | | |  | |  |  | | |  |
| Liberal | 13.7 | 86.3 | |  | | 6.3 | | 93.7 | | |  | | 9.4 | | 90.6 | |  | | 10.5 | | | 89.5 | |  | 7.6 | | | 92.4 |
| Moderate | 25.2 | 74.8 | |  | | 19.4 | | 80.6 | | |  | | 23.6 | | 76.4 | |  | | 12.9 | | | 87.1 | |  | 21.5 | | | 78.5 |
| Conservative | 35.1 | 64.9 | |  | | 30 | | 70 | | |  | | 34.4 | | 65.6 | |  | | 51.8 | | | 48.2 | |  | 27.1 | | | 72.9 |
| Don’t know/ prefer not to answer | 42.4 | 57.6 | |  | | 25.4 | | 74.6 | | |  | | 25.4 | | 74.6 | |  | | 43.5 | | | 56.5 | |  | 36.7 | | | 63.3 |
| **I trust that my government is able to deliver the COVID-19 vaccine to everyone, everywhere in my country, equally** | | | | | | | | | | | | | | | | | | | | | | | | | | |  |  |
| Age |  |  |  | |  | |  | |  |  | |  | |  | |  | |  | |  |  | |  | | |  |  |  |
| 18-29 | 34.3 | 65.7 | |  | | 36.3 | | 63.7 | | |  | | 40.3 | | 59.7 | |  | | 27.9 | | | 72.1 | |  | 42 | | | 58 |
| 30-39 | 38.1 | 61.9 | |  | | 32.7 | | 67.3 | | |  | | 25.8 | | 74.2 | |  | | 31.2 | | | 68.8 | |  | 32.2 | | | 67.8 |
| 40-49 | 29.9 | 70.1 | |  | | 28.6 | | 71.4 | | |  | | 16.8 | | 83.2 | |  | | 25 | | | 75 | |  | 23.3 | | | 76.7 |
| 50-59 | 32.1 | 67.9 | |  | | 18.1 | | 81.9 | | |  | | 23.3 | | 76.7 | |  | | 25.1 | | | 74.9 | |  | 34.1 | | | 65.9 |
| 60-69 | 26.6 | 73.4 | |  | | 16.2 | | 83.8 | | |  | | 15.4 | | 84.6 | |  | | 32.4 | | | 67.6 | |  | 22.8 | | | 77.2 |
| 70+ | 18.4 | 81.6 | |  | | 11.9 | | 88.1 | | |  | | 10.6 | | 89.4 | |  | | 26 | | | 74 | |  | 17.4 | | | 82.6 |
| Sex |  |  | |  | |  | |  | | |  | |  | |  | |  | |  | | |  | |  |  | | |  |
| Male | 29.3 | 70.7 | |  | | 27.7 | | 72.3 | | |  | | 21.8 | | 78.2 | |  | | 31.1 | | | 68.9 | |  | 27.9 | | | 72.1 |
| Female | 31 | 69 | |  | | 21.5 | | 78.5 | | |  | | 24.8 | | 75.2 | |  | | 23.9 | | | 76.1 | |  | 29.2 | | | 70.8 |
| Race |  |  | |  | |  | |  | | |  | |  | |  | |  | |  | | |  | |  |  | | |  |
| White | 31 | 69 | |  | | 31.5 | | 68.5 | | |  | | 22.5 | | 77.5 | |  | | 40.6 | | | 59.4 | |  | 26.9 | | | 73.1 |
| Black | 33.3 | 66.7 | |  | | 19.4 | | 80.6 | | |  | | 30.6 | | 69.4 | |  | | 20.4 | | | 79.6 | |  | 32.7 | | | 67.3 |
| Latino/a | 27.1 | 72.9 | |  | | 19.9 | | 80.1 | | |  | | 19.7 | | 80.3 | |  | | 13.6 | | | 86.4 | |  | 28.8 | | | 71.2 |
| Asian | 26.2 | 73.8 | |  | | 14 | | 86 | | |  | | 31.2 | | 68.8 | |  | | 16.2 | | | 83.8 | |  | 31 | | | 69 |
| Highest level of education |  |  | |  | |  | |  | | |  | |  | |  | |  | |  | | |  | |  |  | | |  |
| High school degree or less | 29.9 | 70.1 | |  | | 18.3 | | 81.7 | | |  | | 22.2 | | 77.8 | |  | | 26.1 | | | 73.9 | |  | 23.1 | | | 76.9 |
| Some college | 30.9 | 69.1 | |  | | 36.9 | | 63.1 | | |  | | 22.3 | | 77.7 | |  | | 29.3 | | | 70.7 | |  | 33.4 | | | 66.6 |
| Bachelor's Degree | 31.3 | 68.7 | |  | | 24.4 | | 75.6 | | |  | | 28.7 | | 71.3 | |  | | 27.9 | | | 72.1 | |  | 36.4 | | | 63.6 |
| Graduate degree or more | 29.3 | 70.7 | |  | | 26.3 | | 73.7 | | |  | | 23.1 | | 76.9 | |  | | 30.2 | | | 69.8 | |  | 28.6 | | | 71.4 |
| Household income |  |  | |  | |  | |  | | |  | |  | |  | |  | |  | | |  | |  |  | | |  |
| < $25,000 | 35.7 | 64.3 | |  | | 12.2 | | 87.8 | | |  | | 33.5 | | 66.5 | |  | | 25.2 | | | 74.8 | |  | 26.5 | | | 73.5 |
| $25,000-$74,999 | 31.1 | 68.9 | |  | | 28.7 | | 71.3 | | |  | | 13.5 | | 86.5 | |  | | 24.3 | | | 75.7 | |  | 36.1 | | | 63.9 |
| $75,000-$150,000 | 23.7 | 76.3 | |  | | 21.7 | | 78.3 | | |  | | 31.3 | | 68.7 | |  | | 27.4 | | | 72.6 | |  | 27 | | | 73 |
| >$150,000 | 30.5 | 69.5 | |  | | 29 | | 71 | | |  | | 27.5 | | 72.5 | |  | | 25.6 | | | 74.4 | |  | 37 | | | 63 |
| Prefer not to say | 36.2 | 63.8 | |  | | 31.4 | | 68.6 | | |  | | 25.4 | | 74.6 | |  | | 44.4 | | | 55.6 | |  | 20.9 | | | 79.1 |
| Employment |  |  | |  | |  | |  | | |  | |  | |  | |  | |  | | |  | |  |  | | |  |
| Working from home | 26.9 | 73.1 | |  | | 30.1 | | 69.9 | | |  | | 27.1 | | 72.9 | |  | | 21.8 | | | 78.2 | |  | 34.4 | | | 65.6 |
| Working outside the home | 38.3 | 61.7 | |  | | 24.9 | | 75.1 | | |  | | 23.8 | | 76.2 | |  | | 32.4 | | | 67.6 | |  | 33.4 | | | 66.6 |
| Not employed | 27.9 | 72.1 | |  | | 20.9 | | 79.1 | | |  | | 21 | | 79 | |  | | 28.5 | | | 71.5 | |  | 21.3 | | | 78.7 |
| Political views |  |  | |  | |  | |  | | |  | |  | |  | |  | |  | | |  | |  |  | | |  |
| Liberal | 24.7 | 75.3 | |  | | 24.2 | | 75.8 | | |  | | 27.1 | | 72.9 | |  | | 22.1 | | | 77.9 | |  | 26.1 | | | 73.9 |
| Moderate | 29.6 | 70.4 | |  | | 20.1 | | 79.9 | | |  | | 23.1 | | 76.9 | |  | | 16.9 | | | 83.1 | |  | 28.7 | | | 71.3 |
| Conservative | 33.1 | 66.9 | |  | | 33.2 | | 66.8 | | |  | | 26.8 | | 73.2 | |  | | 44.8 | | | 55.2 | |  | 32.9 | | | 67.1 |
| Don’t know/ prefer not to answer | 45.2 | 54.8 | |  | | 28.6 | | 71.4 | | |  | | 15.8 | | 84.2 | |  | | 42.2 | | | 57.8 | |  | 36.4 | | | 63.6 |

**Supplemental Table S4. Vaccination requirements and documentation**

|  | National | Metropolitan area | | | | National vs. metropolitan area comparison | | | | | |  |  |
| --- | --- | --- | --- | --- | --- | --- | --- | --- | --- | --- | --- | --- | --- |
|  |  |  |  |  |  | NY | LA | | Dallas | | Chicago |  |  |
|  |  | NY | LA | Dallas | Chicago |  |  |  |  |  |  |  |  |
|  | % | % | % | % | % | p-value | | | | | |  |  |
| **Do you believe that employers have the right to require that people take a COVID-19 vaccine?** | | | | | | <.0001 | | .021 | | .192 | .041 |  |  |
| Unsure/disagree/strongly disagree | 42.3 | 27.5 | 32.9 | 37.9 | 34.3 |  | |  | |  |  |  |  |
| Strongly agree/agree | 57.7 | 72.5 | 67.1 | 62.1 | 65.7 |  | |  | |  |  |  |  |
| **Do you believe that the government should require people to take a COVID-19 vaccine?** | | | | | | <.0001 | | .001 | | .243 | .002 |  |  |
| Unsure/disagree/strongly disagree | 50 | 37.5 | 37.2 | 46 | 37.9 |  | |  | |  |  |  |  |
| Strongly agree/agree | 50 | 62.5 | 62.8 | 54 | 62.1 |  | |  | |  |  |  |  |
| **Do you believe that universities should require students to take a COVID-19 vaccine?** | | | | | | <.0001 | | .038 | | .330 | <.001 |  |  |
| Unsure/disagree/strongly disagree | 37.7 | 23.1 | 30.1 | 34.5 | 24.6 |  | |  | |  |  |  |  |
| Strongly agree/agree | 62.3 | 76.9 | 69.9 | 65.5 | 75.4 |  | |  | |  |  |  |  |
| **Should proof of vaccination be required to enter places like movie theaters, concerts, and sports arenas?** | | | | | | <.0001 | | .009 | | .330 | .031 |  |  |
| Unsure/disagree/strongly disagree | 47.7 | 29.3 | 37.1 | 44.3 | 39 |  | |  | |  |  |  |  |
| Strongly agree/agree | 52.3 | 70.7 | 62.9 | 55.7 | 61 |  | |  | |  |  |  |  |
| **Should proof of vaccination be required for international travel?** | | | | | | <.0001 | | <.001 | | .716 | .001 |  |  |
| Unsure/disagree/strongly disagree | 30.4 | 16 | 19.6 | 31.6 | 20.4 |  | |  | |  |  |  |  |
| Strongly agree/agree | 69.6 | 84 | 80.4 | 68.4 | 79.6 |  | |  | |  |  |  |  |
| Note. n, % are weighted to the geographic population; p-value based on weighted chi-squared tests. | | | | | | | | | | | | | |

**Supplemental Table S5. Sources of information on the vaccine and top priorities for the country by vaccination status**

|  | National | | Metropolitan area | | | | | | | | | | | | | |  |
| --- | --- | --- | --- | --- | --- | --- | --- | --- | --- | --- | --- | --- | --- | --- | --- | --- | --- |
|  |  | | | NY | | | LA | | | Dallas | | | Chicago | | |  |  |
|  | % | | | % | | | % | | | % | | | % | | |  |  |
| **Source of information on the vaccine** | | | | | | | | | | | | | | | | |  |
| The Centers for Disease Control and Prevention (CDC) | 41.8 | | | 45.6 | | | 47.6 | | | 38.6 | | | 44.3 | | |  |  |
| The World Health Organization (WHO) | 10.9 | | | 10.2 | | | 11.1 | | | 13.1 | | | 10 | | |  |  |
| The Federal government | 4.4 | | | 6.6 | | | 4.2 | | | 5.7 | | | 3.6 | | |  |  |
| The state government | 4.1 | | | 5.3 | | | 4 | | | 4.5 | | | 3.2 | | |  |  |
| The city government | 3 | | | 5.1 | | | 3.9 | | | 3.9 | | | 5.2 | | |  |  |
| Community leader | 2.7 | | | 1.3 | | | 1.6 | | | 1.6 | | | 3.8 | | |  |  |
| Faith leader | 1.8 | | | 2.1 | | | 2.5 | | | 1.7 | | | 1.1 | | |  |  |
| My doctor or health care provider | 20.4 | | | 16 | | | 13.5 | | | 18.7 | | | 16.6 | | |  |  |
| Other | 10.8 | | | 7.8 | | | 11.6 | | | 12.2 | | | 12.1 | | |  |  |
| **Top priority for the country right now** | | | | | | | | | | | | | | | | |  |
| Getting people back to work | 18.9 | | | 15.2 | | | 11.4 | | | 17.8 | | | 14.1 | | |  |  |
| Getting all children back to school in person | 12.7 | | | 11.7 | | | 10 | | | 13.7 | | | 13.5 | | |  |  |
| Getting everyone vaccinated as quickly as possible | 42.1 | | | 47.8 | | | 53.8 | | | 47.6 | | | 53.9 | | |  |  |
| Maintaining social distancing and wearing masks | 14.4 | | | 14 | | | 14.8 | | | 8.1 | | | 11.6 | | |  |  |
| Improving access to food, housing, and unemployment aid | 11.9 | | | 11.4 | | | 10.1 | | | 12.7 | | | 6.9 | | |  |  |
| **Top priority for the country right now by vaccination status** | | | | | | | | | | | | | | | |  |  |
| **Vaccine received or planned?** | **No** | **Yes** | | | **No** | **Yes** | | **No** | **Yes** | | **No** | **Yes** | | **No** | **Yes** | | |
| Getting people back to work | 39.7 | 13.2 | | | 32.8 | 13.2 | | 20.5 | 10.2 | | 33.1 | 14.1 | | 45.1 | 10.2 | | |
| Getting all children back to school in person | 19 | 11 | | | 14.2 | 11.4 | | 19.5 | 8.8 | | 27.7 | 10.3 | | 22.1 | 12.4 | | |
| Getting everyone vaccinated as quickly as possible | 13.7 | 49.8 | | | 5.9 | 52.4 | | 14.6 | 58.8 | | 7.2 | 57.5 | | 20.7 | 58.1 | | |
| Maintaining social distancing and wearing masks | 17.8 | 13.5 | | | 24.1 | 12.9 | | 16.8 | 14.5 | | 10.7 | 7.5 | | 2.5 | 12.8 | | |
| Improving access to food, housing, and unemployment aid | 9.9 | 12.4 | | | 22.9 | 10.1 | | 28.6 | 7.7 | | 21.3 | 10.6 | | 9.6 | 6.6 | | |

Note. . % are weighted to the geographic population; Vaccination received or planned: No is Vaccination not received and not planned.

**Supplemental Table S6. Vaccination protection against all variants of COVID-19**

|  | National | Metropolitan area | | | | National vs. metropolitan area comparsion | | | |  |  |
| --- | --- | --- | --- | --- | --- | --- | --- | --- | --- | --- | --- |
|  |  | NY | LA | Dallas | Chicago |  | | | |  |  |
|  | % | % | % | % | % | p-value | | | |  |  |
| **The COVID-19 vaccines will protect you against all variants/types of COVID-19**  **Among respondents vaccinated or those who plan to vaccinate** | | | | | | 0.466 | 0.399 | 0.286 | 0.213 |  |  |
| Unsure/disagree/strongly disagree | 21.3 | 23.5 | 13.9 | 18.5 | 26.6 |  |  |  |  |  |  |
| Strongly agree/agree | 78.7 | 76.5 | 86.1 | 81.5 | 73.4 |  |  |  |  |  |  |
| **Among respondents unwilling to vaccinate** | | | | | | 0.451 | 0.011 | 0.482 | 0.237 |  |  |
| Unsure/disagree/strongly disagree | 88.3 | 82.4 | 79.7 | 91.1 | 94.1 |  |  |  |  |  |  |
| Strongly agree/agree | 11.7 | 17.6 | 20.3 | 8.9 | 5.9 |  |  |  |  |  |  |
| Note. n, % are weighted to the geographic population; p-value based on weighted chi-squared tests. | | | | | | | | | | | |

**Supplemental Figure S1. Approval for vaccination requirements and documentation by a) vaccination status and b) political views**

a) vaccination status

b) political views

#### **Supplemental File S1. Survey instrument in a) English and b) Spanish**

**a) English version**

1. COVID-19 is a dangerous health threat.

a. Strongly agree

b. Somewhat agree

c. Unsure/no opinion

d. Somewhat disagree

e. Strongly disagree

2. COVID-19 can be prevented by vaccination.

a. Strongly agree

b. Somewhat agree

c. Unsure/no opinion

d. Somewhat disagree

e. Strongly disagree

3. The risks of COVID-19 disease are greater than the risks of the vaccine.

a. Strongly agree

b. Somewhat agree

c. Unsure/no opinion

d. Somewhat disagree

e. Strongly disagree

4. The COVID-19 vaccines available to me are safe.

a. Strongly agree

b. Somewhat agree

c. Unsure/no opinion

d. Somewhat disagree

e. Strongly disagree

5. I trust that my government is able to deliver the COVID-19 vaccine to everyone, everywhere in my country, equally

a. Strongly agree

b. Somewhat agree

c. Unsure/no opinion

d. Somewhat disagree

e. Strongly disagree

6. I trust the science behind the COVID-19 vaccines.

a. Strongly agree

b. Somewhat agree

c. Unsure/no opinions

d. Somewhat disagree

e. Strongly disagree

7. Have you received at least one dose of a COVID-19 vaccine?

a. Yes one dose

b. Yes, two doses

c. No (Skip to Q9)

8. Which vaccine did you receive? **(Skip to Q13)**

a. Moderna

b. Pfizer

c. J&J

d. Other

e. Don’t know

9. Please state whether you agree or disagree with the following statement: Now that COVID-19 vaccines are approved for use in the United States by the FDA, I will take a vaccine as soon as it is available to me. **(displayed only if unvaccinated)**

a. Strongly agree (skip to Q13)

b. Agree (skip to Q13)

c. Unsure/ no opinion

d. Disagree

e. Strongly disagree

10. Which of the following would be the top reason for waiting to get the vaccine? (rotate answers)

a. See how it works in other people

b. Let high-risk people go first

c. Wait until it is more convenient

d. Wait until I know there are no serious complications

11. Which of the following would help you feel more ready to take the vaccine? (rotate answers)

a. Assurance by a family member or close friend who got vaccinated

b. Advice from community/religious leaders

c. Recommendation from my family doctor

d. Endorsement from a trusted political leader

e. Nothing will change my mind

f. Something else

12. Where would you most prefer to get the vaccine? (rotate answers)

a. Local pharmacy like CVS or Walgreens

b. Hospital

c. Sports stadium

d. Your doctor’s office

e. Mobile unit send by the department of health to your neighborhood

f. Local schools

g. At a shopping mall or community centers

h. My place of worship (e.g. church, temple, mosque)

i. Somewhere else

13. Which of the following was or would be your main reason to get the vaccine?

a. I want to protect myself from COVID-19

b. I want to protect my friends and family from COVID-19

c. To help end the pandemic more quickly.

d. I need it to get back to work

e. My doctor or health plan/insurance recommended it

f. I will not get the vaccine for any reason

14. Did you have difficulty in making an appointment to get the vaccine?

a. Yes

b. No (skip to Q16)

c. I have not tried to make an appointment (skip to Q16)

15. What difficulty did you have when you made your appointment?

a. I didn’t know how to sign up for an appointment

b. I didn’t know if I was eligible for a vaccine

c. There were no vaccination sites close to me

d. I was worried about the cost

e. I couldn’t take time off the day an appointment was available

f. I don’t have child or dependent care

g. I don’t have access to internet

16. What source for information do you most trust about COVID-19 vaccines?

a. The Centers for Disease Control and Prevention (CDC)

b. The World Health Organization (WHO)

c. The Federal government

d. The state government

e. The city government

f. Community leader

g. Faith leader

h. My doctor or health care provider

i. Other

17. Where do you get most of your information about COVID-19?

a. Radio

b. Cable news

c. Local news

d. Newspapers

e. Social media

f. Internet search

g. Family and friends

h. My religious leader

i. My doctor or health care provider

j. Somewhere else

18. Have you or someone in your household been tested for COVID-19 since the start of the pandemic in March 2020? This would be a nasal or throat swab performed by a medical professional.

a. Yes, I have

b. Yes, a household member

c. Yes, both

d. No (skip to Q18)

19. Was that test positive?

a. Yes, I had a COVID-19 positive test

b. Yes, a household member had a COVID-19 positive test

c. Yes, both

d. No

20. Do you agree or disagree with the following statement? “The information I got about Covid-19 vaccines was clear and easy to understand.”

a. Strongly agree

b. Somewhat agree

c. Unsure/no opinion

d. Somewhat disagree

e. Strongly disagree

21. Next, “People are being pressured by outside sources (e.g., pharmaceutical companies, governments, etc.) to take Covid-19 vaccines.”

a. Strongly agree

b. Somewhat agree

c. Unsure/no opinion

d. Somewhat disagree

e. Strongly disagree

22. Do you believe that employers have the right to require that people take a COVID-19 vaccine?

a. Strongly agree

b. Somewhat agree

c. Unsure/no opinion

d. Somewhat disagree

e. Strongly disagree

23. Do you believe that the government should require people to take a COVID-19 vaccine?

a. Strongly agree

b. Somewhat agree

c. Unsure/no opinion

d. Somewhat disagree

e. Strongly disagree

24. Do you believe that universities should require students to take a COVID-19 vaccine?

a. Strongly agree

b. Somewhat agree

c. Unsure/no opinion

d. Somewhat disagree

e. Strongly disagree

25. Should proof of vaccination be required to enter places like movie theaters, concerts, and sports arenas?

a. Strongly agree

b. Somewhat agree

c. Unsure/no opinion

d. Somewhat disagree

e. Strongly disagree

26. Should proof of vaccination be required for international travel?

a. Strongly agree

b. Somewhat agree

c. Unsure/no opinion

d. Somewhat disagree

e. Strongly disagree

27. How confident are you that the COVID-19 vaccines will protect you against all variants/types of COVID-19?

a. Very confident

b. Somewhat confident

c. Unsure/no opinion

d. Not very confident

e. Not confident at all

28. Would you say your views in most political matters are liberal, moderate, or conservative?

a. Liberal

b. Moderate

c. Conservative

d. Don’t know/ prefer not to answer

29. Are you currently employed?

a. Yes, and I am working from home

b. Yes, and I am working outside the home

c. Not employed

30. From the following list which do you think is the top priority for the country right now? (answers rotated)

a. Getting people back to work

b. Getting all children back to school in person

c. Getting everyone vaccinated as quickly as possible

d. Maintaining social distancing and wearing masks

e. Improving access to food, housing, and unemployment aid

31. What is your age?

32. What is your gender?

a. Male

b. Female

c. Prefer not to say

d. Other

33. What is your race?

a. African American or Black

b. Asian

c. Caucasian/White

d. American Indian or Alaska Native

e. Native Hawaiian or Pacific Islander

f. Hispanic/Latino

g. Multiple/Other

34. What is the highest level of education you have received?

a. High school degree or less

b. Some college

c. Bachelor's Degree

d. Graduate degree or more

35. What is your household income?

a. < $25,000

b. $25,000-$74,999

c. $75,000-$150,000

d. >$150,000

e. Prefer not to say

36. Zip code

**b) Spanish version**

Para empezar, por favor, indique si está de acuerdo o en desacuerdo con las siguientes declaraciones:

1. COVID-19 es una amenaza peligrosa para la salud.

Totalmente de acuerdo

Más o menos de acuerdo

No estoy seguro/no opinión

Más o menos en desacuerdo

Totalmente en desacuerdo

2. Covid-19 se puede prevenir con vacuna

Totalmente de acuerdo

Más o menos de acuerdo

No estoy seguro/no opinión

Más o menos en desacuerdo

Totalmente en desacuerdo

3. Los riesgos de la enfermedad COVID-19 son mayores que los riesgos de la vacuna.

Totalmente de acuerdo

Más o menos de acuerdo

No estoy seguro/no opinión

Más o menos en desacuerdo

Totalmente en desacuerdo

4. Las vacunas COVID-19 disponibles para mí son seguras.

Totalmente de acuerdo

Más o menos de acuerdo

No estoy seguro/no opinión

Más o menos en desacuerdo

Totalmente en desacuerdo

5. Confío en que mi gobierno sea capaz de entregar la vacuna COVID-19 a todos, en todas partes de mi país, igualmente

Totalmente de acuerdo

Más o menos de acuerdo

No estoy seguro/no opinión

Más o menos en desacuerdo

Totalmente en desacuerdo

6. Confío en la ciencia detrás de las vacunas COVID-19.

Totalmente de acuerdo

Más o menos de acuerdo

No estoy seguro/no opinión

Más o menos en desacuerdo

Totalmente en desacuerdo

7. ¿Usted recibió al menos una dosis de una vacuna COVID-19?

Si una dosis

Si, dos dosis

No

8. ¿Qué vacuna recibió?

Moderna

Pfizer

J&J

Otro

No se

Por favor, indique si está de acuerdo o en desacuerdo con la siguiente declaración:

9. Ahora que la FDA aprueba el uso de las vacunas COVID-19 en los Estados Unidos, tomaré una vacuna tan pronto como esté disponible para mí.

Totalmente de acuerdo

Más o menos de acuerdo

No estoy seguro/no opinión

Más o menos en desacuerdo

Totalmente en desacuerdo

10. ¿Cuál de las siguientes sería la principal razón para esperar para recibir la vacuna?

Vea cómo funciona en otras personas

Deje que las personas de alto riesgo vayan primero

Espere hasta que sea más conveniente

Espera a que sepa que no hay complicaciones graves

Garantía de un familiar o amigo cercano que recibió la vacuna

Consejo de líderes comunitarios/religiosos

Recomendación de mi médico de familia

Respaldo de un líder político de confianza

Nada cambiará mi mente

Algo más

12. ¿Dónde prefiere la vacuna?

Farmacia local como CVS e Walgreens

Hospital

Estadio deportivo

El consultorio de su médico

Unidad móvil enviada por el departamento de salud a su vecindario

Escuelas locales

En un centro comercial o centros comunitarios

Mi lugar de culto (por ejemplo, iglesia, templo, mezquita)

En otro lugar

13. ¿Cuál de las siguientes fue o sería su principal razón para recibir la vacuna?

Quiero protegerme de COVID-19

Quiero proteger a mis amigos y familiares de COVID-19

Ayudar a poner fin a la pandemia más rápidamente.

Lo necesito para volver al trabajo

Mi médico o plan de salud/seguro lo recomendó No voy a recibir la vacuna por ninguna razón

14. ¿Tuvo dificultades para hacer una cita para obtener la vacuna?

Sí

No

No he intentado hacer una cita

15. ¿Qué dificultad tuvo cuando hizo su cita?

No sabía cómo inscribirse para una cita

No sabía si era elegible para una vacuna

No había sitios de vacunación cerca de mí

Estaba preocupado por el costo

No pude tomar tiempo libre el día en que una cita estaba disponible

No tengo cuidado de niños o dependientes

No tengo acceso a Internet

16. ¿Qué fuente de información confía más sobre las vacunas COVID-19?

Los Centros para la Prevención y el Control de Enfermedades (CDC)

La Organización Mundial de la Salud (OMS)

El gobierno federal

El gobierno estatal

El gobierno de la ciudad

Líder comunitario

Líder de fe

Mi médico o proveedor de atención médica Otros

17. ¿Dónde puede obtener la mayor parte de su información sobre COVID-19?

Radio

Noticias de cable

Noticias locales

Periódicos

Redes sociales

Búsqueda en Internet

Familia y amigos

Mi líder religioso

Mi médico o proveedor de atención médica En otro lugar

18. ¿Usted o alguien de su hogar ha hecho una prueba de COVID-19 desde el comienzo de la pandemia en marzo de 2020? Esto sería un hisopo nasal o de garganta realizado por un profesional médico.

Sí, he

Sí, un miembro del hogar

Sí, los dos

No

19. ¿Dio positivo?

Sí, tuve una prueba positiva COVID-19

Sí, un miembro del hogar tuvo una prueba positiva COVID-19

Sí, los dos

No

20. vacunas Covid-19 fue clara y fácil de entender".

Totalmente de acuerdo

Más o menos de acuerdo

No estoy seguro/no opinión

Más o menos en desacuerdo

Totalmente en desacuerdo

21. A continuación, “las personas están siendo presionadas por fuentes externas (por ejemplo, compañías farmacéuticas, gobiernos, etc.) para que tomen las vacunas Covid-19”.

Totalmente de acuerdo

Más o menos de acuerdo

No estoy seguro/no opinión

Más o menos en desacuerdo

Totalmente en desacuerdo

22. ¿Cree que los empleadores tienen derecho a exigir que las personas tomen una vacuna COVID-19?

Totalmente de acuerdo

Más o menos de acuerdo

No estoy seguro/no opinión

Más o menos en desacuerdo

Totalmente en desacuerdo

23. ¿Cree que el gobierno debería exigir a la gente que tome una vacuna COVID-19?

Totalmente de acuerdo

Más o menos de acuerdo

No estoy seguro/no opinión

Más o menos en desacuerdo

Totalmente en desacuerdo

24. ¿Cree que las universidades deberían exigir a los estudiantes que tomen una vacuna COVID-19?

Totalmente de acuerdo

Más o menos de acuerdo

No estoy seguro/no opinión

Más o menos en desacuerdo

Totalmente en desacuerdo

25. ¿Debería exigirse una prueba de vacunación para entrar en lugares como salas de cine, conciertos y estadios deportivos?

Totalmente de acuerdo

Más o menos de acuerdo

No estoy seguro/no opinión

Más o menos en desacuerdo

Totalmente en desacuerdo

26. ¿Debería ser necesaria la prueba de vacunación para los viajes internacionales?

Totalmente de acuerdo

Más o menos de acuerdo

No estoy seguro/no opinión

Más o menos en desacuerdo

Totalmente en desacuerdo

27. ¿Qué tan seguro está de que las vacunas COVID-19 le protegerán contra todas las variantes/tipos de COVID-19?

Muy confiado

Un poco confiado

Inseguro/sin opinión.

No estoy muy seguro

No estoy confiado para nada

28. ¿Diría que sus puntos de vista en la mayoría de los asuntos políticos son liberales, moderados o conservadores?

Liberales

Moderados

Conservadores

No se/ prefiero no contestar

29. Está actualmente empleado?

Si, y trabajo desde casa

Si, y trabajo fuera de casa

No estoy empleado

30. De la siguiente lista, ¿cuál cree que es la prioridad principal para el país en este momento?

Conseguir que la gente vuelva al trabajo

Hacer que todos los niños vuelvan a la escuela en persona

Vacunar a todos lo mas rapido que posible

Mantener el distanciamiento social y usar máscaras

Mejorar el acceso a la ayuda alimentaria, a la vivienda y al desempleo

31. Cual es tu edad?

32. Cual es tu género?

Hombre

Mujer

Prefiero no decir

Otro

33. ¿Cuál es tu raza?

Afroamericano o negro

Asiático

Caucásico/Blanco

Índio americano o nativo de Alaska

Nativo hawaiano o isleño del Pacífico

Hispano/Latino

Múltiples/otros

34. ¿Cuál es el nivel más alto de educación que ha recibido?

Escuela secundaria

Alguna universidad

Bachillerato

Posgrado o más

35. ¿Cuál es su ingreso familiar?

$25,000

$25,000-$75,000

$75,000-$150,000 >$150,000

Prefiero no decir

36. Codigo postal
